# Diphtheria Treated by Chloral Hydrate

**Published:** 1888-11

**Authors:** 


Diphtheria Treated by Chloral Hydrate.—Dr. Mercier (*Rev. de Thérap.*, Feb.,) reports very good results. Before giving chloral, if the tongue be much furred, he administers an emetic—preferably ipecacuanha, in powder. He then gives from one and a-half to five grains of chloral, in the form of a syrup, every half hour, taking care to give food and drink beforehand, so as to leave the syrup in contact with the throat. The administration of liquids before the chloral prevents the latter giving rise to gastric pain. The drug generally stopped the further progress of the disease, and within forty-eight hours the false membranes disappeared, and the raw surface left was gargled with an astringent lotion. The treatment is only of use in the early stages of the disease, and is without benefit when the larynx has become involved.

---

DR. COOKE, in the *Medical Record*, adduces observations to show that when conception occurs early in the night, boys result; when in the morning, girls. We may justly infer that conceptions occurring in the middle of the night will result in hermaphrodites.

---

THE JEWISH CONSISTORY OF PARIS has appointed a committee to consult with surgeons as to the best method of performing *peritomy* (circumcision). It is proposed to appoint an *inspector of peritomy*. Dr. Duplay, an eminent surgeon, has been delegated to write a manual describing the best method of performing the operation.

---

ALL REJECTED.—All the candidates appearing before the British Columbia Examining Board were rejected this year. It should be said that the *all* included *two* candidates only.—*The Journal.*

---

THE NEW YORK BOARD OF HEALTH now requires that all plumbing be inspected, and tested by the air-pump and pressure-gauge, in the same manner as now employed in testing gas-pipes.

---

MONTEFUSCO, PHYSICIAN TO THE COTUGNO SMALL-POX HOSPITAL, recommends phenic acid, locally and internally, as the best treatment for variola.

---

DR. JULIAN J. CHISHOLM, of Baltimore, has recently successfully transplanted a rabbit's cornea into the human eye, the third successful case on record.

---

OF THE PARIS OPHTHALMOLOGISTS, DeWelcker is a German, Galezowski a Pole, Landolt a Swiss, Panas a Greek, and Abadie French.

---

MAGISTRATE—"Have you no written document to prove that your wife is really dead?" Peasant—"I have the doctor's bill."—*Fliegende Blatter.*

